# Social Support and Adherence to Treatment Regimens among Patients Undergoing Hemodialysis

**DOI:** 10.3390/healthcare12191958

**Published:** 2024-10-01

**Authors:** Amnah A. Alatawi, Marym Alaamri, Hayfa Almutary

**Affiliations:** 1 Registered Nurse (RNs), King Fahad Specialist Hospital, Tabuk 32253, Saudi Arabia; 2 Medical Surgical Nursing Department, Faculty of Nursing, King Abdulaziz University, Jeddah 21589, Saudi Arabia

**Keywords:** adherence, hemodialysis, nonadherence, treatment, social support

## Abstract

Background/Objectives: Following recommended treatment plans is essential to the success of the treatment. In hemodialysis, adherence to the treatment regimen remains a challenge in many healthcare settings. Many factors, including the patient’s level of social support, influence treatment adherence, but few studies have addressed these relationships. Methods: A correlational cross-sectional design was used to assess the relationship between perceived social support and adherence to treatment regimens among patients undergoing hemodialysis. Self-reported surveys were used to collect data. Results: One hundred and twenty-one hemodialysis patients were recruited from two dialysis centers. Overall, 45.5% of participants demonstrated good adherence, 47.9% had moderate adherence, and 6.6% had poor adherence. Specifically, 76% of participants consistently took their prescribed medications, 75% regularly attended hemodialysis sessions, 67.8% adhered to dietary restrictions, and 62% followed fluid-intake guidelines. Perceived social support was reported as highest from family members (89.3%), followed by significant others (62.8%) and friends (49.6%). There were significant correlations between perceived social support and overall adherence to treatments among hemodialysis patients. Conclusions: This study provides evidence for positive relationships between perceived social support and adherence to treatment regimens among hemodialysis patients. It is recommended that professional caregivers intervene with clients and their families, prepare recreational and educational programs for patients undergoing hemodialysis, and inspire other researchers to suggest different social support models and approaches.

## 1. Introduction

Managing end-stage kidney disease (ESKD) requires dialysis therapy or kidney transplantation for survival [1]. Although kidney transplantation is the best choice for the treatment of ESKD, it is limited by the availability of organ donors, their physical or mental state, and patients’ preferences and decisions [2,3]. Hemodialysis (HD) comprises 89% of dialysis used worldwide [4]. It is a complex treatment method requiring regular, continuous visits to a dialysis unit, typically three times per week. In addition, an effective and successful HD treatment requires many lifestyle changes, including the use of multiple medications, restricted food and fluid intake, and strict adherence to treatment regimens. Consequently, risks of nonadherence are high [5].

Nonadherence to treatment regimens typically includes not taking the required medications, missing or reducing HD sessions, or the overconsumption of fluids and foods containing potassium and phosphorus [6]. A recent systematic review and meta-analysis of 23 studies showed poor adherence to diet and fluid restrictions among HD patients, ranging between 47.3% and 72.5% for diet and 50% and 70.7% for fluids [7].

Nonadherence to HD requirements is a major concern because it can result in life-threatening complications, including increased morbidity and mortality, as well as spiraling costs for both the patient and the healthcare system [8]. Associated complications include cardiovascular risks, fatal arrhythmias, hypervolemia, and osteodystrophy [2,5].

Many factors influence a patient’s adherence. Social support is critical in treatment outcomes for many chronic diseases, including ESKD [9]. Social support involves activities or relationships within social structures, providing affection, care, or a sense of attachment to a trusted social group or partner [10]. HD patients often experience significant reductions in social contact due to dialysis schedules, chronic fatigue, or psychological issues such as depression. Assistance and encouragement from social support resources promotes patients’ adherence, by fostering confidence and self-esteem, buffering against the stresses of being ill, minimizing depression, and enhancing sick-role behavior [11].

Few studies have investigated the relationship between nonadherence to treatment and social support [12,13,14,15,16]. To our knowledge, there are no previous data on the relationship between social support and adherence to treatment regimens among HD patients in Saudi Arabia. Therefore, further exploration in this area is essential to identify gaps in adherence, enabling healthcare practitioners to implement appropriate interventions and reduce the health and economic impacts of nonadherence.

## 2. Materials and Methods

### 2.1. Design

This is a quantitative cross-sectional correlational study. A correlational design examines the relationships between two phenomena that either coincide or result from one another.

### 2.2. Participants

This study was conducted in two hemodialysis units at two major hospitals in Saudi Arabia’s North Region using convenience nonprobability sampling. Adults over the age of 18 undergoing HD who could communicate in either English or Arabic were eligible. Patients with mental or cognitive impairments and those on peritoneal dialysis were excluded. The appropriate sample size was calculated using the online Raosoft calculating program, with an error margin of 5% at a confidence level of 95%. Of the 176 patients at the two dialysis units, 121 were recruited, based on these calculations and the above criteria.

### 2.3. Data Collection

The data were gathered in December 2021 during a period of three months. A researcher met the participants in the HD unit waiting areas before their sessions or inside the units while they were on dialysis. The researcher explained the study’s purpose to the participants who met the inclusion criteria and decided to participate, and informed them that they could withdraw from the study at any time without penalty or impact on the management of their care. Each participant was provided written informed consent that clearly stated the purpose of the study, procedures, confidentiality and privacy, and potential risks and benefits.

Self-reported surveys were used to collect data. Participants were given the option of completing a 30–35 min survey, either in the dialysis unit or at home. The first researcher helped participants who needed assistance to complete the questionnaire by reading the questionnaire questions aloud without giving an explanation in the same manner, thereby ensuring the prevention of potential bias. Before conducting the study, we addressed all ethical obligations and obtained permission from the ethics committees of the Faculty of Nursing (reference no. 1M. 13) and from the two hospitals (IRB no. TU-077/020/056). Permissions to use research tools were also secured from their authors.

### 2.4. Measures

The study questionnaires included sociodemographic information, and two standardized instruments related to the main study variables. The sociodemographic parameters taken were age, gender, marital status, educational level, employment status, and monthly income. The end-stage renal disease adherence questionnaire (ESRD-AQ) was used to assess HD patients’ adherence in this study [17]. The scale comprises forty-six questions and items that measure five dimensions. The first part of the scale seeks general knowledge on the experiences of patients with ESKD and renal replacement therapy (five items), while the other four parts focus on HD, which includes treatment adherence (fourteen items), medications (nine items), fluid restriction (ten items), and diet recommendations (eight items). The ESRD-AQ responses use a combination of Likert scale, multiple choice, and ’yes’ or ‘no’ response formats. Adherence behavior was scored by summing responses to selected questions (item 14, 17, 18, 26, 31 and 46) with scores weighted by degree of importance of each dimension to the relevant clinical outcomes. Higher ESRD-AQ scores indicate better adherence [17]. Table 1 shows the interpreting scores for ESRD-AQ.

The scale employed for this study proved both valid and reliable, exhibiting strong test-retest stability across every item of the ESRD-AQ, and showed intra-class correlation (ICC) scores ranging from 0.83 to 1.00 [17]. Cronbach’s alpha values ranged from 0.70 to 0.798 [18,19]. The Arabic version of ESRD-AQ was also valid and reliable. The Cronbach’s alpha value of the Arabic version was 0.72 [20]. Cronbach’s alpha was 0.700 in the current study.

The multidimensional scale of perceived social support (MSPSS) was also used to determine everyone’s perceived social support elements [21]. The scale comprises 12 items and uses a 7-point Likert-type scale from ‘strongly disagree’ to ‘strongly agree’. The MSPSS measures an individual’s perception of social support by examining three specific resources: family, friends, and significant other. Each subscale includes four components. To calculate the mean score on the scale, an average of all 12 items is obtained and then divided by 12. However, the highest and lowest scores that are obtainable from the scale are 84 and 12, respectively. The score is then categorized into three levels: low perceived social support for scores ranged between 1–2.9, medium perceived social support for scores ranged between 3–5, and high perceived social support for scores ranged between 5.1–7. The validity and reliability of both English and Arabic versions of the MSPSS have been demonstrated previously with Cronbach’s alphas ranging from 0.87 to 0.95 [16,21,22,23]. In the current study, the Cronbach’s alpha value for the MSPSS was 0.93.

### 2.5. Data Analysis

Data were coded and analyzed using SPSS software, Version 21. Statistical significance was accepted at *p* < 0.05. Descriptive statistics were calculated for participants’ demographic characteristics, level of adherence to treatment regimens, and level of perceived social support. Fisher’s exact test was used to examine the relationship between perceived social support and overall adherence to treatment regimens among HD patients.

## 3. Results

### 3.1. Sociodemographic Characteristics of the Participants

One hundred and twenty-one HD patients were enrolled in this study. One-third of the participants were 18–40 years old (Table 2) and more than half were males (56.2%). Thirty-eight percent were high school graduates, with fourteen percent having bachelor’s or higher degrees. Approximately half were married or not working (both 49.6%).

### 3.2. Level of Adherence to Treatment Regimens among Patients Undergoing HD

**Figure 1:**
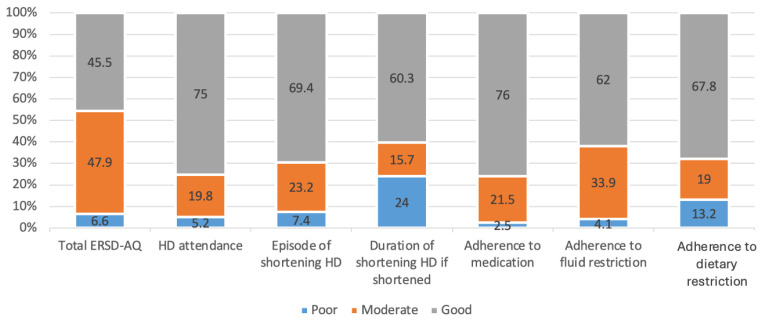
Level of adherence to treatment regimens based on ESRD-AQ.

Adherence was good amongst all four categories (dietary, fluid, medications, and attendance to dialysis sessions) as presented in Figure 1. Medication intake showed the highest adherence, at 76%, followed by HD attendance (75%), then diet restriction (67.8), with fluid restriction being the lowest, at 62%. The overall adherence was good among 45.5% of patients; 47.9% were moderate and only 6.6% were poor.

### 3.3. Level of Perceived Social Support among Patients Undergoing HD

**Figure 2:**
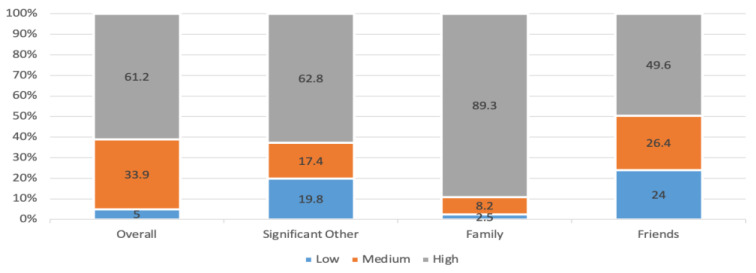
Level of perceived social support and its domains based on MSPSS.

Figure 2 presents the level of perceived social support among patients undergoing HD. Perceived social support was highest in ‘family (89.3%), followed by significant other (62.8%) and friends (49.6%). The overall perceived social support was high among 61.2% of patients, medium among 33.9% and low among 5%.

### 3.4. Relationship between Overall Adherence to Treatment Regimens, Perceived Social Support and Sociodemographic Characteristics of the Participants

Good adherence to treatment regimens was statistically significant amongst patients 60 years or older (*p* = 0.026) and those who were unemployed (*p* = 0.050). There were no other significant relationships between sociodemographic parameters (i.e., gender, nationality, educational level, marital status, and monthly income) and adherence to treatment (Table 3). No significant relationships were identified between levels of perceived social support and any sociodemographic factors (Table 3).

### 3.5. Relationship between Perceived Social Support and Overall Adherence to Treatment among Patients Undergoing HD

There was a significant relationship between high perceived social support among patients undergoing HD and overall adherence to their treatment regimens (*p* = 0.019), (see Table 4).

## 4. Discussion

Treatment adherence is a major challenge to the effective management of patients receiving HD. Estimating the prevalence of adherence and assessing its causes are crucial to understanding the impact of dialysis treatment on a patient’s lifestyle. We found that adherence to all aspects of treatment was good, although there was an association between high perceived levels of social support and higher overall adherence to treatment regimens. Patients on HD are strictly instructed to follow a diet low in sodium, potassium, and phosphorus and to maintain an adequate protein intake while limiting their daily fluid intake [24]. One could argue that adherence to dietary restrictions is one of the most difficult lifestyle modifications required in HD treatment regimens [25], although in the present study two-thirds of patients adhered to such restrictions. This figure is higher than the 46.6% and 37% adherence reported in Indian and African-American populations, respectively [25,26]. However, our findings contrasted with a report from a recent systematic review and meta-analysis that showed a global prevalence of nonadherence to diet and fluid restrictions of approximately 60% [7]. The present study’s results may have been due to the dietary counselling all participants received. Luitel et al. (2020) found that respondents who received health information from a dietitian had a high level of adherence to these dietary guidelines. In addition, most of the participants in our sample are in the middle-age group, which may explain their commitment to dietary restriction [27]. Prior studies found that adherence to dietary restrictions is positively associated with increased age [28,29].

Conversely, only 4.1% of our participants showed poor adherence to fluid restrictions, possibly because this study was conducted during the winter season, when fluid consumption may naturally be lower. This result is consistent with a prior study which found that nonadherence to fluid restrictions impacted 4% of the participants [13]. Another possible explanation for our finding is that fluid restrictions may be easier to manage in a country that bans alcohol, such as the one in our study. Social drinking, which sometimes results in intoxication and encourages further drinking, is a large part of the cultures of many non-Muslim countries, which makes these comparisons difficult. More studies may be needed to assess seasonal fluid restriction adherence and cultural differences.

Many individuals undergoing HD are prescribed nine to eighteen pills a day for comorbidities, in addition to the medications required for ESKD [25], which add challenge to medication adherence. A systematic review of studies from 1970 to 2014 reported that the prevalence of medication nonadherence varied from 12.5% to 98.6% [30]. Interestingly, our study found that only 2.5% of participants had poor adherence to prescription medication, with the majority adhering well (76%). These findings are similar to those of several other studies [31,32,33]. This result may be influenced by the support of the healthcare system in Saudi Arabia, which provides 100% coverage for all prescribed medications. Insufficient insurance and low income are associated with patients’ medication nonadherence in chronic diseases [34,35]. However, subjective self-reports may also influence the results. Our study is based on subjective self-reports (using the ESRD-AQ scale) that measure the overall perception of the individuals about their adherence to taking medications. It asks the patient how often they have missed their prescribed medicines. Therefore, we did not collect objective data, such as a follow-up chart showing the number of prescribed medications taken daily, to confirm adherence. However, a future study could potentially collect both subjective and objective data to assess adherence by reviewing and counting all medications taken by the patient.

Missed treatment sessions and those shortened by more than 10 min are considered indicators of nonadherence [16]. The study showed that three-quarters (75%) of patients went to scheduled HD sessions, with 5% showing poor attendance, which reflects good HD adherence. Adherence in this study was slightly higher than that reported in the other reports [2,19,28]. This high rate of attendance is likely due to the free access to dialysis therapy in Saudi Arabia for ESKD patients, and the compatibility of dialysis with patients’ schedules. A recent study showed that patients with health insurance coverage less than 1% and financial constraints contributed to a 21% nonadherence rate to HD sessions within that population [1]. Moreover, there is sufficient support from the Saudi government for this population to secure their rights and help them alleviate their suffering and disabilities [36]. For example, the employees with kidney failure are granted a fully paid leave for the days they undergo HD [36]. In addition, the ministry of health provides free transportation services for those who have transportation issues. Moreover, it is the responsibility of medical and nursing staff in HD units to educate their patients and emphasize the importance of HD appointments so that patients do not miss or shorten them, and to routinely follow up with patients with poor attendance to determine the reason for missed sessions.

Regarding overall patient adherence, the findings indicate that less than half (45.5%) of the participants undergoing HD had good levels of adherence to the therapeutic regimen, almost half (48%) had moderate overall levels of adherence, and only 6.6% had poor adherence. This is due to the tendency of patients to adhere more to one component of their therapeutic regimen than others. Prior studies have shown similar overall adherence to treatment regimens [2,18,37].

A patient’s sociodemographic profile is a vital predictor of treatment adherence. Overall levels of adherence were significantly higher among patients who were more than 60 years old. This is consistent with previous reports [9,28,38] and can potentially be explained by older patients having more structured lifestyles that can more easily accommodate the demands of the treatment routine. Patients who are younger may believe that they are less susceptible to unfavorable health consequences [28]. Moreover, the study revealed that levels of adherence were significantly higher among unemployed patients with more time to attend therapy. However, several studies found that employed patients were significantly more likely to adhere to treatment regimen [23,39]. Usually, the employment status reflects in some way the financial status, which is an important factor that influences treatment adherence in general. The full coverage of treatment costs in Saudi Arabia for this population may be the logical rationale for our finding. However, more studies, with larger sample sizes and different socioeconomic statuses, may be needed to investigate this phenomenon.

Patients undergoing HD treatment have identified their families as their most important sources of support. In this study, patients’ social support levels were found to be high. When subgroups of the scale were evaluated, the results showed that perceived support levels from family members were higher than those from significant others such as partners, healthcare providers, and friends. This is consistent with a systematic review of social support and treatment adherence in patients with ESKD [15], which revealed that social support, especially in the form of family, significant others, and health professionals, can increase treatment adherence in patients with ESKD undergoing dialysis. We believe these results may be due to the importance placed on family support in Saudi culture.

This study showed that about half of the participants reported moderate or low support from friends. This may be due to a decreased participation among this group in community activities and in interactions with friends. This result is consistent with Ahrari et al. (2014), who also found that HD patients receive low levels of support from friends [9]. Again, this is likely due to patients with ESKD experiencing significant reductions in social interaction because of their treatment schedule and associated symptoms [15].

This study found that high perceived levels of social support among patients undergoing HD resulted in significantly higher adherence to their treatment regimens. This result is also consistent with Ahrari et al. (2014), whose Iranian study reported a significant relationship between social support and adherence to dietary and fluid restrictions [9]. These findings are also supported by Varghese (2018) who found a statistically significant relationship between perceived social support and treatment adherence [19]. Furthermore, the study conducted by Miyata et al. (2018) showed that Japanese patients had stronger social support, and that this family support might contribute to Japanese patients’ positive attitudes toward, and thus adherence to, dialysis treatment [40].

The majority of participants in this study were Saudi Arabian, so its findings may be influenced by Saudi culture and society, which values close family ties even during illnesses to maintain a positive social image [41]. Saudi families place great importance on caring for older relatives suffering from chronic diseases, with family members contributing to recovery efforts [41]. In addition, this study’s findings may be influenced by the fact that more than half of the participants were married, potentially providing additional support for treatment adherence [28,38].

Social support may benefit patients by providing encouragement, empathy, warmth, and hope, increasing motivation to adhere to treatment protocols [13]. Effective support from family members can encourage patients to better adjust to ESKD and dialysis requirements by decreasing distress, depression, feelings of loneliness, and perceived disease burden, while also facilitating disease acceptance, healthcare service utilization, and problem-solving skills [15].

This study’s findings have important implications for clinical practice in the field of HD. The study highlights the significance of perceived levels of social support in positively influencing adherence to treatment among HD patients. Healthcare professionals should recognize this influence and take steps to enhance and encourage supportive networks for their patients, and should involve family members, significant others, and friends in the treatment process. By equipping healthcare providers with the tools to educate patients about the importance of adherence and supporting patients’ social engagement, they can indirectly improve adherence rates. Furthermore, healthcare professionals can also play a role in fostering social support networks within the dialysis center. Organizing support groups or educational sessions where patients can connect with others undergoing similar treatments can create a sense of community and provide opportunities for peer support. Additionally, healthcare providers should regularly assess the levels of perceived social support among their patients. By understanding each patient’s support network, healthcare professionals can tailor their interventions and strategies to address any gaps or barriers to adherence. Future longitudinal and experimental studies are needed to investigate social support and adherence among peritoneal and pre-dialysis patients and to fully understand the associations among these variables.

There are certain limitations in this study. Determining the causality between adherence and social predictors in our findings is challenging because this was a cross-sectional study. The findings should be cautiously projected onto Saudi dialysis patients because the convenience sample of the study was small, which minimizes the generalizability of the findings. Moreover, we cannot exclude the possibility of recall bias due to the use of self-reported questionnaires.

## 5. Conclusions

Adherence to treatment regimens that require significant lifestyle modifications is challenging for patients undergoing HD. However, this study supports the existing evidence for the positive relationship between perceived levels of social support and adherence. We hope that this study will guide healthcare professionals’ interventions with patients and their families, help them prepare recreational and educational programs for their patients, and encourage other researchers to suggest different approaches and models for social support.

## Figures and Tables

**Table 1:** Interpreting scores for end-stage renal disease adherence questionnaire (ESRD-AQ).

Item Number in ESRD-AQ	Adherence	Score	Poor	Moderate	Good
14	HD—attendance	0–300	<200	200–<250	250–300
17	Episode of shortening HD	0–200	<100	100–<150	150–200
18	Duration of shortening HD if shortened	0–100	<50	50–<75	75–100
26	Adherence to medication	0–200	<100	100–<150	150–200
31	Adherence to fluid restriction	0–200	<100	100–<150	150–200
46	Adherence to dietary restriction	0–200	<100	100–<150	150–200

**Table 2:** Demographic characteristics of the participants (*n* = 121).

Study Variables	N (%)
Age group	
18–40 years	38 (31.4%)
41–50 years	29 (24.0%)
51–60 years	23 (19.0%)
>60 years	31 (25.6%)
Mean, SD	48.74 ± 17.07
Gender	
Male	68 (56.2%)
Female	53 (43.8%)
Educational level	
Not educated	22 (18.2%)
Elementary school	26 (21.5%)
Intermediate	10 (08.3%)
High school	46 (38.0%)
Bachelor or higher	17 (14.0%)
Marital status	
Single	31 (25.6%)
Married	60 (49.6%)
Divorced or widowed	30 (24.8%)
Occupational status	
Employed	26 (21.5%)
Unemployed	60 (49.6%)
Student	08 (06.6%)
Retired	27 (22.3%)
Monthly income (SR)	
<3000 SR	67 (55.4%)
≥3000 SR	54 (44.6%)

**Table 3:** Relationship between overall adherence to treatment regimens and sociodemographic characteristics (*n* = 121).

Factor	Level of Adherence to Treatment Regimens			*p*-Value ^§^	Level of Perceived Social Support			*p*-Value ^§^
	Poor N (%) (*n* = 8)	Moderate N (%) (*n* = 58)	Good N (%) (*n* = 55)		Low N (%) (*n* = 6)	Medium N (%) (*n* = 41)	High N (%) (*n* = 74)	
**Age group**								
18–40 years	04 (50.0%)	22 (37.9%)	12 (21.8%)	0.026 **	03 (50.0%)	14 (34.1%)	21 (28.4%)	0.260
41–50 years	02 (25.0%)	14 (24.1%)	13 (23.6%)		01 (16.7%)	08 (19.5%)	20 (27.0%)	
51–60 years	02 (25.0%)	13 (22.4%)	08 (14.5%)		01 (16.7%)	12 (29.3%)	10 (13.5%)	
>60 years	0	09 (15.5%)	22 (40.0%)		01 (16.7%)	07 (17.1%)	23 (31.1%)	
**Gender**								
Male	04 (50.0%)	30 (51.7%)	34 (61.8%)	0.505	04 (66.7%)	19 (46.3%)	45 (60.8%)	0.275
Female	04 (50.0%)	28 (48.3%)	21 (38.2%)		02 (33.3%)	22 (53.7%)	29 (39.2%)	
**Educational level**								
Not educated	01 (12.5%)	10 (17.2%)	11 (20.0%)	0.335	01 (16.7%)	09 (22.0%)	12 (16.2%)	0.754
Elementary school	01 (12.5%)	11 (19.0%)	14 (25.5%)		01 (16.7%)	10 (24.4%)	15 (20.3%)	
Intermediate	01 (12.5%)	08 (13.8%)	01 (01.8%)		01 (16.7%)	03 (07.3%)	06 (08.1%)	
High school	04 (50.0%)	19 (32.8%)	23 (41.8%)		01 (16.7%)	15 (36.6%)	30 (40.5%)	
Bachelor or higher	01 (12.5%)	10 (17.2%)	06 (10.9%)		02 (33.3%)	04 (09.8%)	11 (14.9%)	
**Marital status**								
Single	03 (37.5%)	17 (29.3%)	11 (20.0%)	0.336	02 (33.3%)	11 (26.8%)	18 (24.3%)	0.679
Married	02 (25.0%)	26 (44.8%)	32 (58.2%)		03 (50.0%)	17 (41.5%)	40 (54.1%)	
Divorced or widowed	03 (37.5%)	15 (25.9%)	12 (21.8%)		01 (16.7%)	13 (31.7%)	16 (21.6%)	
**Occupational status**								
Employed	02 (25.0%)	13 (22.4%)	11 (20.0%)	0.050 **	02 (33.3%)	09 (22.0%)	15 (20.3%)	0.770
Unemployed	05 (62.5%)	27 (46.6%)	28 (50.9%)		03 (50.0%)	23 (56.1%)	34 (45.9%)	
Student	01 (12.5%)	07 (12.1%)	0		0	01 (02.4%)	07 (09.5%)	
Retired	0	11 (19.0%)	16 (29.1%)		01 (16.7%)	08 (19.5%)	18 (24.3%)	
**Monthly income (SAR)**								
<3000	05 (62.5%)	35 (60.3%)	27 (49.1%)	0.443	04 (50.0%)	23 (56.1%)	40 (54.1%)	0.863
≥3000	03 (37.5%)	23 (39.7%)	28 (50.9%)		02 (33.3%)	18 (43.9%)	34 (45.9%)	

^§^ *p*-value has been calculated using Fisher’ exact test. ** Significant at *p* ≤ 0.05 level.

**Table 4:** Relationship between perceived social support and overall adherence to treatment regimens (*n* = 121).

MSPSS	ESRD-AQ			*p*-Value ^§^
	Poor N (%) (*n* = 8)	Moderate N (%) (*n* = 58)	Good N (%) (*n* = 55)	
Low perceived support	02 (25.0%)	04 (06.9%)	0	0.019 **
Medium perceived support	03 (37.5%)	22 (37.9%)	16 (29.1%)	
High perceived support	03 (37.5%)	32 (55.2%)	39 (70.9%)	

^§^ *p*-value has been calculated using Fisher’ exact test. ** Significant at *p* ≤ 0.05 level.

## Data Availability

The datasets used and analyzed during the current study are available from the corresponding author on reasonable request.
